# Gnaphalieae (Asteraceae): diversity and distribution in Rio de Janeiro – Brazil

**DOI:** 10.3897/BDJ.13.e142891

**Published:** 2025-05-21

**Authors:** Marcelo de Oliveira Gigier, Gustavo Heiden, Rafaela Campostrini Forzza

**Affiliations:** 1 Jardim Botânico do Rio de Janeiro, Rio de Janeiro, Brazil Jardim Botânico do Rio de Janeiro Rio de Janeiro Brazil; 2 Embrapa Clima Temperado, Pelotas, Brazil Embrapa Clima Temperado Pelotas Brazil; 3 Instituto Chico Mendes de Conservação da Biodiversidade, Prado, Brazil Instituto Chico Mendes de Conservação da Biodiversidade Prado Brazil

**Keywords:** Atlantic Rainforest, distribution range, geographical patterns, Grasslands, Restinga, rock outcrop vegetation

## Abstract

**Background:**

As an area of great diversity and suffering from many threats, the Brazilian *Mata Atlântica* and its floristic zones, such as *Restingas*, Rock Outcrops and Grasslands, call for attention and recurrent studies on plant diversity as a means of helping conservation efforts. In this context, acquiring, curating and using herbaria data is crucial to filling gaps in plant distribution and biogeography, as well as confirming or denying species incidence in the area of interest. This study provides a solid dataset with information regarding diversity and distribution of species from the tribe Gnaphalieae (Asteraceae) in the State of Rio de Janeiro, Brazil, with significant updates since the publication of Flora Fluminensis by Vellozo almost 200 years ago, the Rio de Janeiro checklist one decade ago and the milestone of the *Flora e Funga do Brasil* continuously updated dynamic dataset.

**New information:**

We recorded 31 species of Gnaphalieae (Cass.) Lecoq. & Juill. for Rio de Janeiro State of which nine are newly recorded in the area and five are refuted occurrences, compared to the listing in *Flora e Funga do Brasil* 2023. In our compilation, we confirmed the occurrence of eight genera in the State flora: *Achyrocline* (Less.) DC., *Chevreulia* Cass., *Chionolaena* DC., *Facelis* Cass., *Gamochaeta* Wedd., *Gnaphalium* L., *Lucilia* Cass. and *Pseudognaphalium* Kirp. Moreover, we compared the cost-benefit on using municipality centroids versus original/curated coordinates on 5 km^2^ quadrants. We found that, on this geographical scale, there is no significant difference between the two methods. We advocate for using the less time-consuming centroids, which are less prone to human error, for expediting presence/absence checklist data curation and if the main goals are to quantify records, map species richness and evaluate sampling effort. Nevertheless, precise coordinates are essential for ecological niche modelling, conservation assessments and other data usage, focusing on habitat level mapping.

## Introduction

The *Mata Atlântica* domain stands out as one of the world’s biodiversity hotspots (Myers et al. 2000). Although mostly known for its forests, the domain comprises additional small and diverse areas: the Grasslands (*Campos de Altitude* and Rock Outcrops) and the *Restingas* (Coastal scrubs) (IBGE 2004, Brasil 2006). Due to its biodiversity and the multiple threats it suffers by human occupation, agriculture and other changes in land cover (Ribeiro et al. 2009), it is important to use herbarium information to maintain an accurate dataset for monitoring the occurrences of the taxa known to the area. This is mainly because knowing taxa geographic distribution can contribute to conservation actions (Albani Rocchetti et al. 2021), especially extinction risk evaluation and establishment of new protected areas (Giam et al. 2010), as it allows tracking species distributions in relation to changes in land use and coverage across time. Therefore, the State of Rio de Janeiro stands out as a candidate study area, as it is a relatively small State, entirely embedded in the Mata Atlântica, spanning an area of 43,750 km² (IBGE 2021) and has important areas of grasslands and rock outcrops as well as Restingas with the associated occurrence of Asteraceae (Roque et al. 2017).

Gnaphalieae is one of the largest tribes in Asteraceae, comprising 2,106 species (Mandel et al. 2019). The tribe is readily recognised by its dry, papery involucral bracts that can be enlarged and showy, usually with the typical strap-shaped ligule of the ray floret being absent (Smissen et al. 2020). The tribe remains largely understudied in Brazil, mostly being known on the southernmost part of the country (i.e. Deble and Marchiori (2014a), Deble and Marchiori (2014b)), despite some authors indicating south-eastern Brazil as one of the key diversity areas for the tribe (Dillon and Sagástegui 1991), that is also a global biodiversity Darkspot recently recognised by Ondo et al. (2024). Flora e Funga do Brasil (Deble et al. 2023) indicates the occurrence of seven genera and 27 species in Rio de Janeiro, a surprising 36% of all species known in the country (total of 71, Deble et al. (2023)), mostly associated with Restingas and Grasslands.

Despite being one of the most floristically well sampled and studied State, especially considering Vellozo's pioneer efforts (Vellozo 1881, Bediaga and Lima 2015) and the Rio de Janeiro Flora checklist (Baumgratz et al. 2014), the Gnaphalieae genera and species in south-eastern Brazil, specifically in *Mata Atlântica* and, overall, in Rio de Janeiro, have been inadequately studied. Thus, the quality of the data available is questionable, especially regarding presence/absence of species and distribution data. In this study, we gather data and propose to discuss three topics: 1) to summarise the genera and species of Gnaphalieae confirmed by voucher specimens occurring in Rio de Janeiro as a mean to update *Flora e Funga do Brasil* (Deble et al. 2023); 2) to identify the geographical ranges of the tribe in the State and 3) to quantify sampling efforts and evaluate the cost-benefit of two geographic coordinate data inputs. For our third goal, we confronted the usage of centroids with accurate coordinates (informed by authors on the collection voucher or interpreted when traceable specific localities were given in labels) as a means of contributing to filling gaps in species distribution (Wheeler et al. 2012, Hortal et al. 2015) and improving the quality of herbaria data available for species richness and biogeography (Lavoie 2013).

## Sampling methods

### Sampling description

We started from the genera of Gnaphalieae indicated in *Flora e Funga do Brasil* (Deble et al. 2023, Flora e Funga do Brasil - Lista Oficial 2023) as native to the State of Rio de Janeiro. Then, a dataset of geographic distribution records, based on specimens of all sampled species, was built to analyse the distribution range. The data were obtained from the following online biodiversity repositories: GBIF (2022), JABOT (2023) and speciesLink (2023). The collections found in the herbaria: ALCB, B, BC, BHCB, BR, CAP, CAS, CEN, CEPEC, CESJ, CPMA, EAC, ECT, ESA, ESAL, F, FCAB, FUEL, FURB, G, GH, HB, HBR, HCF, HRB, HRCB, HRJ, HUEFS, HUEMG, HUENF, HUFU, HUNI, IBGE, ICN, INPA, IPA, K, L, M, MA, MBM, MG, MNHN, MO, MPEG, NY, P, PEL, PEUFR, PMSP, R, RB, RBR, RFA, RFFP, S, SI, SJRP, SP, SPF, UB, UEC, UFG, UFP, UPCB, US, VAL, VIC, VIES and W Herbaria (Thiers 2023) were consulted in person or online.

Distribution points were inferred, based on localities and verified using Google Earth (2024), as each occurrence was associated with the centroid coordinate of the municipality cited on the collection voucher. As a matter of comparison, we then carefully curated each point for the most detailed coordinate possible (when the original coordinate was unavailable or erroneous, such as points in the sea or out of the State's terrestrial borders). We obtained 151 unique coordinates, then we measured the distance between the centroid and the original point, as well as the mean distance for the 117 altered coordinates on the entire dataset (Table 1). All specimens considered in the dataset were verified by a specialist and revised or nomenclaturally updated when necessary. The final dataset contains 942 unique records, from filtering 1,808 initial occurrences and 176 additional records that cannot be assigned to a specific municipality or locality in the State.

**Figure 1.**
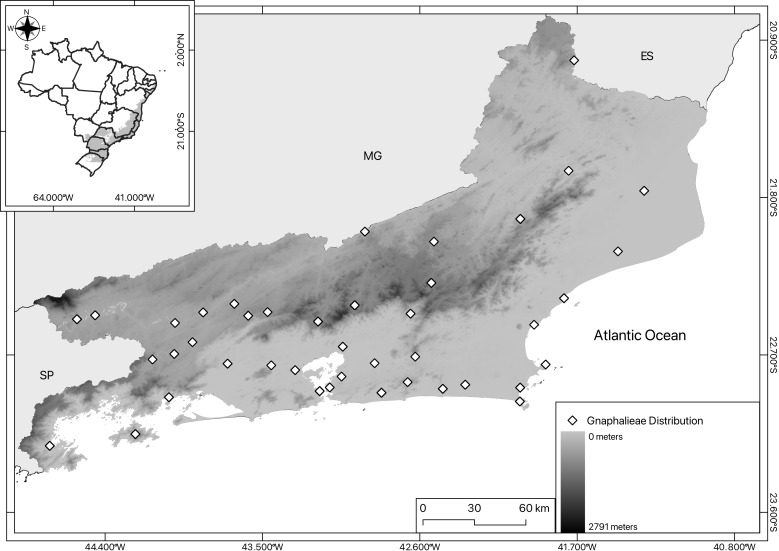
Overview of geographical distribution for eight genera (*Achyrocline*, *Chevreulia*, *Chionolaena*, *Facelis*, *Gamochaeta*, *Gnaphalium*, *Lucilia* and *Pseudognaphalium*) of Gnaphalieae in Rio de Janeiro State considering the State's elevation (varying from 0 at its lowest point to 2,791 m at the Agulhas Negras peak in Parque Nacional do Itatiaia).

The map (Fig. 1) was created using QGIS (Quantum GIS Development Team 2024) by plotting the collection localities on the Brazilian base map from IBGE (2020).

**Sampling effort estimation**: Rio de Janeiro State was divided in 5 km × 5 km quadrants, in which both the centroid and curated points were plotted, in separate maps (Fig. 2). The total number of sampled species for each coordinate was calculated by the sum of total points occurring in a quadrant and displayed as a heatmap.

**Figure 3.**
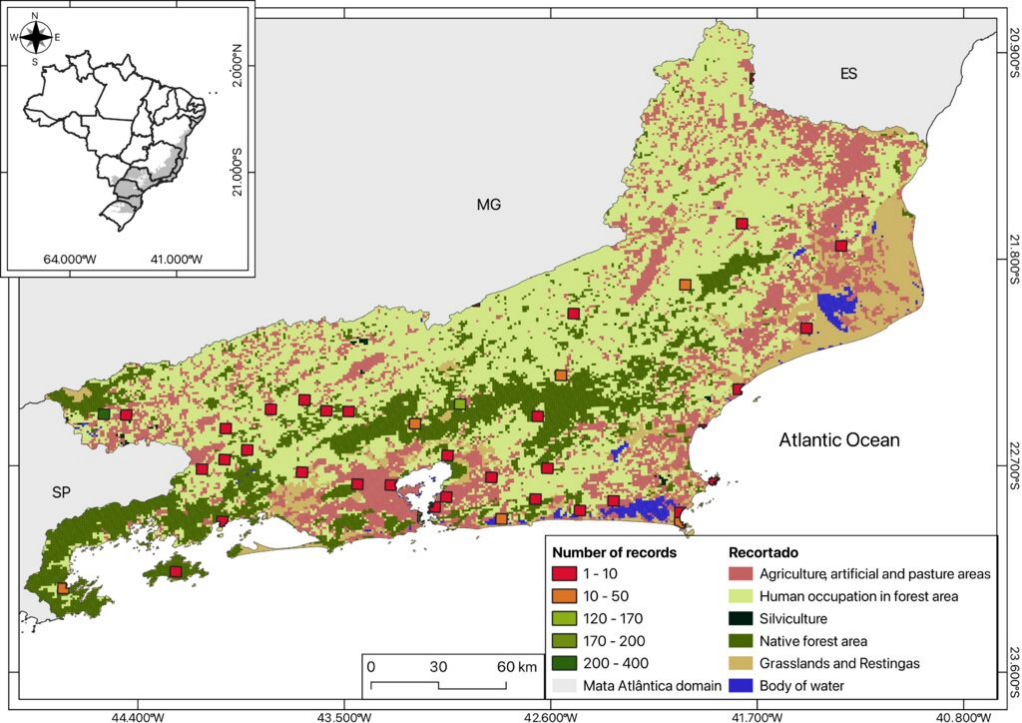
Heatmap (quantifying sampling efforts) using centroid coordinates on Land use and coverage data of Rio de Janeiro State, south-eastern Brazil.

The maps (Figs 1, 2, 3) were created using QGIS (Quantum GIS Development Team 2024) by plotting the total number of collected specimens per locality on the Brazilian base map from IBGE (2020) as well as the land use and coverage from IBGE (2024).

## Geographic coverage

### Description

The geographic coverage encompasses 44 out of 92 municipalities in the Rio de Janeiro State with occurrence points, with Itatiaia being the most well-sampled (384 occurrences) and with several municipalities presenting only one occurrence (Duas Barras, Miguel Pereira, São Fidélis etc.). Most of the specimens, as observed, occurred in Grasslands, either Campos de Altitude or Rock outcrops (Fig. 3) (Veloso and Filho 1982). More than half of the States' municipalities (48) do not have any Gnaphalieae records, even though some native species belonging to the tribe are of widespread occurrence, commonly cultivated for folk medicine, or can be ruderal or even weedy.

### Coordinates

−23° and −20° Latitude; −44° and −42° Longitude.

## Taxonomic coverage

### Description

We recorded the occurrence of 31 species of Gnaphalieae, distributed in eight genera for Rio de Janeiro, which are the following: *Achyroclinealata* (Kunth) DC., *A.arrojadoana* Mattf., *A.chionaea* (DC.) Deble & Marchiori, *A.flaccida* (Weinm.) DC., *A.gardneri* (Baker) Deble & Marchiori, *A.lanosa* (Wawra) Deble, *A.satureioides* (Lam.) DC., *A.vargasiana* DC., *A.vauthieriana* DC., *Chevreuliaacuminata* Less., *Chionolaenaarbuscula* DC., *C.capitata* (Baker) Freire, *C.isabellae* Baker, *C.latifolia* (Benth.) Baker, *C.lychnophorioides* Sch.Bip., *C.phylicoides* (Gardner) Baker, *C.wittigiana* Baker, *Facelisretusa* (Lam.) Sch.Bip., *Gamochaetaamericana* (Mill.) Wedd., *G.grazielae* (Rizzini) Deble, *G.pensylvanica* (Willd.) Cabrera, *G.purpurea* (L.) Cabrera, *G.simplicicaulis* (Willd. ex Spreng.) Cabrera, *G.stachydifolia* (Lam.) Cabrera, *Gnaphaliumpolycaulon* Pers., *Luciliaferruginea* Baker, *L.linearifolia* Baker, *L.lycopodioide*s (Less.) S.E.Freire, *L.tomentosa* Wedd., *Pseudognaphaliumcheiranthifolium* (Lam.) Hilliard & Burtt and *P.gaudichaudianum* (DC.) Anderb. (Table 2).

The *Flora e Funga do Brasil* (Deble et al. 2023) recognises the occurrence of 27 species and eight genera of the tribe. However, based on our analyses, we confirmed the occurrence of nine new species records and could not prove the occurrence of five species previously associated with the State (Table 2), accounting for a total of four more species than previously thought. Although most of the specimens had a municipality associated with its collection voucher, a minority of our data was incomplete regarding collection site (i.e., municipality, locality or any description of its place of origin), with the only information being the state/province of collection (Rio de Janeiro). In these cases, we considered the occurrence in the State valid, but not detailed enough for geographic coordinate comparison. Moreover, as the State and its capital are homonyms, it is common to find records probably coming from the State capital missing the information of the State itself, leaving a dubious interpretation if the specimen comes from the municipality or any other area of the State.

Amongst these genera, *Achyrocline* and *Chevreulia* have a wide distribution throughout the entire State (Suppl. material 1). More so, *Achyroclinesatureioides*, *Chionolaenacapitata* and *Pseudognaphaliumcheiranthifolium* are the most well-documented species for the area. Conversely, many of the genera have a low number of records and are mostly represented by old specimens, as in the case of *Luciliantomentosa*, which is only known by its type and three other samples with no information regarding date of collection.

Most species are found in the high-altitude Grasslands, which is expected as it is known to harbour a large diversity of Asteraceae. However, the *Restinga*, a coastal open habitat favourable for Asteraceae, which has a similar floristic composition (Araujo 2000, Vasconcelos 2014) was poorly represented (Fig. 3, Suppl. material 1, Table 2), accounting for 102 samples, roughly 9% of the points in our maps. The lack of specimens from 48 municipalities is striking, especially considering the absence of records of common and widespread species. This may represent a complete absence of sampling in these areas or maybe the fact that some botanists avoid sampling herbaceous and ruderal species, hindering the distribution records of species perceived as having less aesthetic, conservation or ecological value.

## Temporal coverage

### Notes

**Figure 4.**
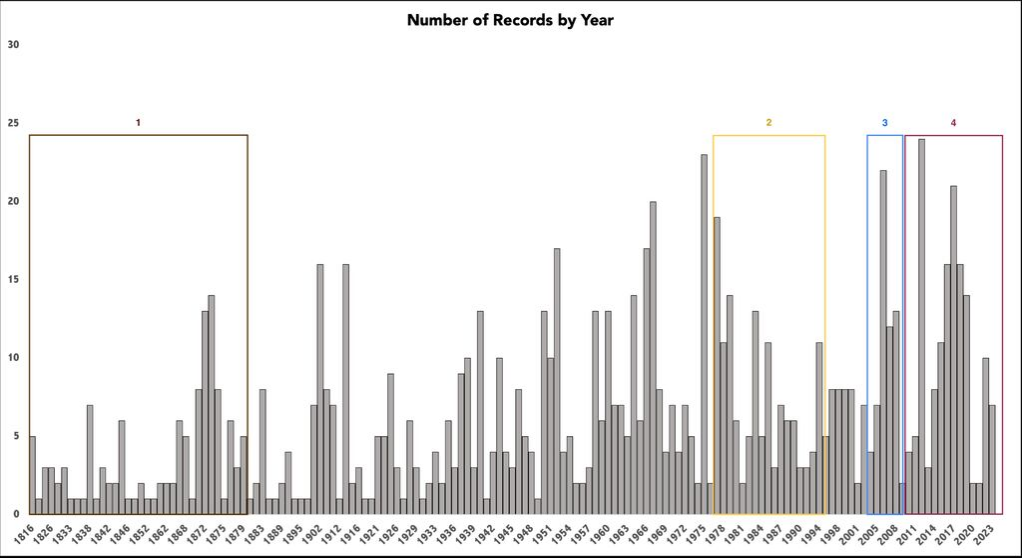
Records of sampled specimens per year in Rio de Janeiro State, south-eastern Brazil.

Our dataset covers collections from 1816 to 2023 (207 years) (Fig. 4), which coincides with when the State flora first started to be surveyed by Frei José Mariano da Conceição Vellozo, although the specimens studied by him had unknown or imprecise collection localities (Bediaga and Lima 2015). Almost two centuries since this pioneer effort, the current list represents a major update for the knowledge of Gnaphalieae in the State's flora. We could identify defined marks in the tribe’s sampling: 1) those in the monarchy era (pre-1881) associated with European naturalists’ presence in Brazil, brought by the Royal Family; 2) the 80s and 90s, especially after the Rio-92 convention for biodiversity, resulted in a spike in the number of sampled species and scientific studies regarding the family in the State; 3) post 2006, with the law enforcing Mata Atlântica conservation efforts, the number of studies comprising the State and Asteraceae were high (e.g. regional Floras and biogeography studies) and 4) close to 2014, when the Flora do Rio project was completed as a functional list, containing various degrees of information regarding the species occurring in the State and their conservation status.

## Usage licence

### Usage licence

Other

### IP rights notes

CC-BY 4.0

## Data resources

### Data package title

Diversity and distribution of Gnaphalieae in Rio de Janeiro – Brazil

### Resource link

 https://zenodo.org/records/14224611

### Number of data sets

1

### Data set 1.

#### Data set name

Distribution and Diversity of Gnaphalieae in Rio de Janeiro

#### Data format

CSV

#### Description

Occurrences of eight genera and 31 species of Gnaphalieae in the Rio de Janeiro State. The set includes 942 entries with coordinates. The dataset was compiled by the usage of data available from herbarium collections on biodiversity repositories online through Rstudio and Excel and contains information on taxonomy, herbarium code, collector, year/month/day of record sample, municipality, locality and geographical coordinates.

**Data set 1.** 

Column label	Column description
Barcode	Herbarium voucher Barcode (used on speciesLink or Reflora).
catalogNumber	Herbarium Catalogue Number.
herbariumCode	Acronym of the herbarium according to Thiers (2023, continuously updated).
year	Indicates year collection of sample.
month	Indicates month collection of sample.
day	Indicates day collection of sample.
municipality	Municipality where the sampling was carried out.
locality	Description of the location where the sampling was carried out.
floweringStatus	If the specimen is in flower or not.
dataOrigin	Repository from where the data were obtained, either speciesLink (spLink), GBIF or REFLORA (RB).
decimalLat.interpreted	Latitude curated and accurate (as of author’s voucher or description).
decimalLong.interpreted	Longitude curated and accurate (as of author’s voucher or description).
decimalLat.centroid	Latitude assigned to centroid.
decimalLong.centroid	Longitude assigned to centroid.
scientificName.FFBR	Full scientific name with author as of Flora e Funga do Brasil.
originalFFB.RG	Indicates if the species is originally assigned on Flora e Funga do Brasil.
recordedBy	Indicates specimen collector.
recordNumber	Indicates the collector personal number for the specimen.
Habitat	If the species are found in Grasslands or Restinga.
date	Full date regarding the format Year-Month-Day (YYYY-MM-DD).
forGeoref	If the specimen data was used for georeferencing and had geographical data.
taxonRank	Regarding if the specimen is identified at the genus or species level.
stateProvince	Indicates the Brazilian State from where the sample originates.

## Additional information

**Conclusion and Prospects**

The dataset herein represents an important step in refining the knowledge of Gnaphalieae for Rio de Janeiro, which is a very representative State for the tribe in the *Mata Atlântica* of southeast Brazil. This dataset includes a total of 31 species, belonging to eight genera. In addition to the remarkable diversity of the group in the area, our study also highlights the challenges faced by most of the species, which is the lack of sampling in more than half of the State municipalities and refined geographic data.

Notably, one of the main foci of our study was to investigate whether assigning the most accurate possible or the centroid coordinate would make a difference to biodiversity and sampling mapping. Thereby, we showed that when the scale is too large (as in the case of Rio de Janeiro, which is a small State by Brazilian standards and roughly the size of Belgium), it does not change the location of the quadrant significantly, with a mean deviation of the original point to the centroid being 13 km (Table 1). Therefore, we measured the deviation and it is visible and plausible that using the centroid instead of the author’s assigned or interpreted coordinate is the most cost-efficient and reliable method, as human-led mistakes (e.g. wrong interpretation of a location or faults in GPS usage) are less prone to happen.

Overall, nine new species were already recorded in the State from herbarium data, but not recognised by Flora e Funga do Brasil and five species recorded in Flora and Funga do Brasil had no herbarium specimens that corroborated their occurrence. This increase is significant and may help future studies to give more attention to these “newly” recorded species and to validate the absence data of previously listed species.

## Supplementary Material

957E01FB-B7D5-54A1-8614-30AF7581A22F 10.3897/BDJ.13.e142891.suppl1 Supplementary material 1 Gnaphalieae distribution maps for each of the eight genera Data type Images Brief description Figures 1 to 5 display the distribution of each of Gnaphalieae's genera in the State of Rio de Janeiro, considering the State's elevation. Figures 3 to 5 are joint distributions for genera that occurrences do not overlap. File: oo_1141600.zip https://binary.pensoft.net/file/1141600 Gigier, M.O.; Heiden, G. and Forzza, R.C.

## Figures and Tables

**Figure 2a.**
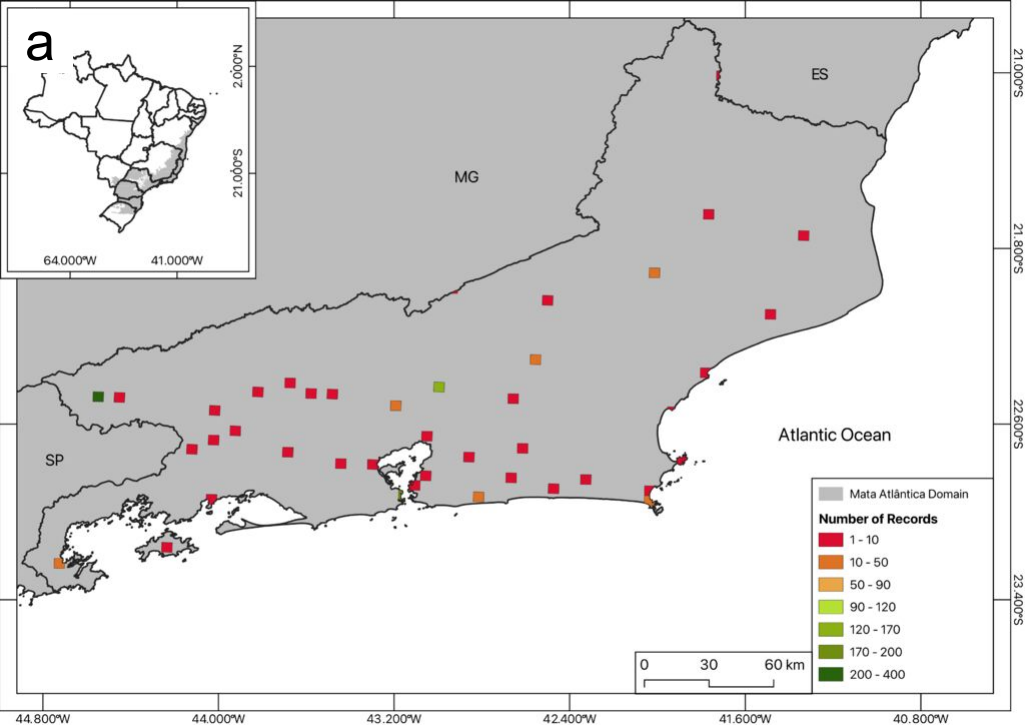


**Figure 2b.**
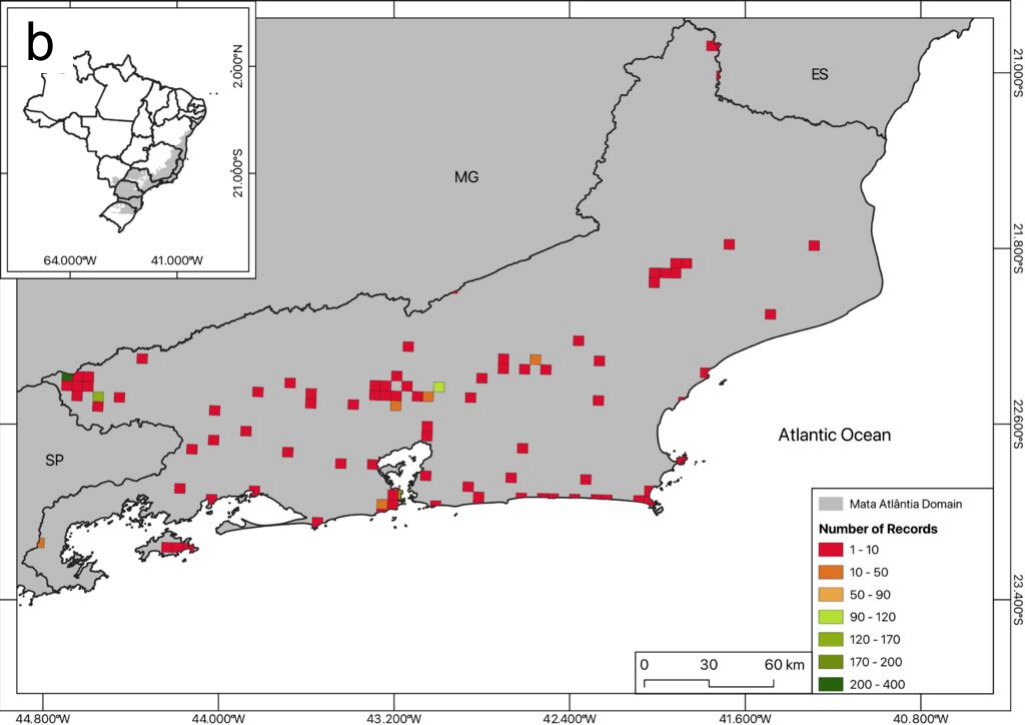


**Table 1.** Distance from the curated to the centroid coordinate, measured in kilometres. The distance of each unique point from the curated to the centroid coordinate was measured using QGIS distance matrix function, the mean distance deviation being measured for the entire dataset.

**Latitude** **interpreted**	**Longitude** **interpreted**	**Latitude** **centroid**	**Longitude** **centroid**	**Municipality**	**Locality**	**Deviation (Km)**
-22.9391	-42.2222	-22.87	-42.3401	Araruama		14.3132
-22.9491	-42.0719	-22.966	-42.0278	Arraial do Cabo		4.8946
-22.9327	-42.2555	-22.966	-42.0278			23.6425
-20.8975	-41.757	-21.0152	-41.7174	Bom Jesus do Itabapoana		13.6666
-20.8763	-41.7247	-21.015239	-41.717415			15.4015
-22.3455	-42.7238	-22.4639	-42.6527	Cachoeiras de Macacu	Parque Estadual dos Três Picos	15.0166
-22.3438	-42.7227	-22.4639	-42.6527		Parque Estadual dos Três Picos	15.1269
-22.3436	-42.7219	-22.4639	-42.6527		Parque Estadual dos Três Picos	15.1074
-22.3205	-42.7227	-22.4639	-42.6527		Parque Estadual dos Três Picos	17.4390
-21.7648	-41.3081	-21.7614	-41.3167	Campos dos Goytacazes		0.96591
-21.7605	-41.6544	-21.7614	-41.3167			34.9303
-22.4999	-44.5633	-22.4957	-44.5609	Itatiaia		0.5265
-22.4961	-44.5633	-22.4957	-44.5609			0.2509
-22.496	-44.5633	-22.4957	-44.5609			0.2491
-22.425	-44.691944	-22.4957	-44.5609		Parque Nacional do Itatiaia	15.5953
-22.423	-44.5913	-22.4957	-44.5609		Parque Nacional do Itatiaia	8.6372
-22.3992	-44.6703	-22.4957	-44.5609		Parque Nacional do Itatiaia	15.5243
-22.3986	-44.6686	-22.4957	-44.5609		Parque Nacional do Itatiaia	23.3046
-22.3855	-44.6797	-22.4957	-44.5609		Parque Nacional do Itatiaia	17.2764
-22.3853	-44.6792	-22.4957	-44.5609		Parque Nacional do Itatiaia	17.2556
-22.3851	-44.6795	-22.4957	-44.5609		Parque Nacional do Itatiaia	17.2931
-22.3848	-44.6794	-22.4957	-44.5609		Parque Nacional do Itatiaia	17.3094
-22.3848	-44.6793	-22.4957	-44.5609		Parque Nacional do Itatiaia	17.3022
-22.3844	-44.6583	-22.4957	-44.5609		Parque Nacional do Itatiaia	15.8881
-22.3842	-44.6797	-22.4957	-44.5609		Parque Nacional do Itatiaia	17.3784
-22.3842	-44.6683	-22.4957	-44.5609		Parque Nacional do Itatiaia	16.5735
-22.3842	-44.694	-22.4957	-44.5609		Parque Nacional do Itatiaia	18.4440
-22.3827	-44.6655	-22.4957	-44.5609		Parque Nacional do Itatiaia	16.5083
-22.3825	-44.666389	-22.4957	-44.5609		Parque Nacional do Itatiaia	16.5848
-22.3819	-44.6647	-22.4957	-44.5609		Parque Nacional do Itatiaia	16.5221
-22.3802	-44.6886	-22.4957	-44.5609		Parque Nacional do Itatiaia	18.3411
-22.3797	-44.7025	-22.4957	-44.5609		Parque Nacional do Itatiaia	19.4289
-22.3761	-44.6661	-22.4957	-44.5609		Parque Nacional do Itatiaia	17.1081
-22.3743	-44.7011	-22.4957	-44.5609		Parque Nacional do Itatiaia	19.7238
-22.3741	-44.7011	-22.4957	-44.5609		Parque Nacional do Itatiaia	19.7390
-22.3741	-44.6743	-22.4957	-44.5609		Parque Nacional do Itatiaia	17.8213
-22.3727	-44.703	-22.4957	-44.5609		Parque Nacional do Itatiaia	19.9878
-22.372	-44.6452	-22.4957	-44.5609		Parque Nacional do Itatiaia	16.2157
-22.3716	-44.6101	-22.4957	-44.5609		Parque Nacional do Itatiaia	14.6460
-22.3715	-44.617	-22.4957	-44.5609		Parque Nacional do Itatiaia	14.9168
-22.3712	-44.6156	-22.4957	-44.5609		Parque Nacional do Itatiaia	14.8923
-22.3697	-44.6283	-22.4957	-44.5609		Parque Nacional do Itatiaia	15.5828
-22.3691	-44.6435	-22.4957	-44.5609		Parque Nacional do Itatiaia	16.3965
-22.3652	-44.6248	-22.4957	-44.5609		Parque Nacional do Itatiaia	15.8779
-22.6005	-43.03	-22.6527	-43.0405	Magé		5.8805
-22.9033	-44.1644	-22.9416	-44.0349	Mangaratiba	Parque Estadual do Cunhambebe	13.9449
-22.9608	-42.8336	-22.916	-42.8191	Maricá		5.1794
-22.9601	-42.8628	-22.916	-42.8191		Área de Proteção Ambiental de Barra de Maricá	6.6289
-22.9194	-42.8183	-22.916	-42.8191			0.3853
-22.9193	-42.8185	-22.916	-42.8191			0.3705
-22.9155	-42.6394	-22.916	-42.8191			18.4348
-22.9089	-42.8961	-22.916	-42.8191		Refúgio da Vida Silvestre de Maricá	7.9383
-22.8925	-42.854167	-22.916	-42.8191			4.4402
-22.5055	-43.3888	-22.4549	-43.4705	Miguel Pereira		10.1039
-22.9725	-43.0185	-22.8858	-43.1152	Niterói	Parque Estadual da Serra da Tiririca	13.8049
-22.4166	-43.1166	-22.288	-42.534	Nova Friburgo		61.6774
-22.3536	-42.5869	-22.288	-42.534		Parque Estadual dos Três Picos	9.0815
-22.333	-42.4955	-22.288	-42.534			6.3692
-22.3119	-42.2661	-22.288	-42.534			27.7320
-22.2113	-42.3513	-22.288	-42.534		Parque Estadual dos Três Picos	20.6594
-23.1402	-44.813333	-23.21971	-44.7167	Paraty	Parque Nacional da Serra da Bocaina	13.2448
-23.14	-44.8135	-23.21971	-44.7167		Parque Nacional da Serra da Bocaina	13.2723
-22.505	-43.178611	-22.5091	-43.1821	Petrópolis		0.5788
-22.505	-43.178333	-22.5091	-43.1821			0.5969
-22.4628	-43.094	-22.5091	-43.1821		Parque Nacional da Serra dos Órgãos	10.4154
-22.4555	-43.2466	-22.5091	-43.1821		Reserva Biológica Estadual das Araras	8.9044
-22.4475	-43.292222	-22.5091	-43.1821		Reserva Biológica Estadual das Araras	13.2275
-22.4355	-43.2466	-22.5091	-43.1821		Reserva Biológica Estadual das Araras	10.5114
-22.4352	-43.2574	-22.5091	-43.1821		Reserva Biológica Estadual das Araras	11.2706
-22.4197	-43.286944	-22.5091	-43.1821			14.6441
-22.4097	-43.2308	-22.5091	-43.1821			13.4267
-22.4058	-43.2169	-22.5091	-43.1821			12.2874
-22.4002	-43.205	-22.5091	-43.1821			15.5243
-22.2422	-43.1261	-22.5091	-43.1821			30.1128
-22.4722	-44.4649	-22.473315	-44.456965	Resende		0.8259
-22.3877	-44.6761	-22.473315	-44.456965			16.8421
-22.3761	-44.6988	-22.473315	-44.456965			27.1251
-22.3133	-44.363	-22.4733	-44.4569			20.1845
-22.4882	-41.896	-22.5273	-41.9463	Rio das Ostras		6.7477
-23.0537	-43.5428	-22.9068	-43.1728	Rio de Janeiro	Parque Municipal Natural de Grumari	41.2798
-22.9879	-43.279	-22.9068	-43.1728		Parque Nacional da Tijuca	14.1174
-22.9732	-43.2494	-22.9068	-43.1728		Parque Nacional da Tijuca Vista Chinesa	10.7610
-22.9716	-43.2054	-22.9068	-43.1728		Parque Municipal Natural da Catacumba	7.9169
-22.9608	-43.2743	-22.9068	-43.1728		Parque Nacional da Tijuca	12.0064
-22.9028	-43.2075	-22.9068	-43.1728			3.5874
-22.5167	-43.206	-22.9068	-43.1728			43.3342
-21.9559	-42.009	-21.9566	-42.0077	Santa Maria Madalena		0.1550
-21.9222	-42.025	-21.9566	-42.0077			4.2075
-21.9186	-41.9516	-21.9566	-42.0077			7.1619
-21.905	-41.9496	-21.9566	-42.0077		Parque Estadual do Desengano	8.2870
-21.9042	-41.9488	-21.9566	-42.0077		Parque Estadual do Desengano	8.4080
-21.8997	-41.9111	-21.9566	-42.0077			11.8025
-21.8988	-41.9119	-21.9566	-42.0077		Parque Estadual do Desengano	11.7865
-21.865556	-41.901111	-21.9566	-42.0077		Parque Estadual do Desengano	14.9307
-21.8494	-41.8702	-21.9566	-42.0077		Parque Estadual do Desengano	18.5141
-21.865556	-41.901111	-21.6466	-41.7489	São Fidélis		28.9081
-22.9278	-42.4511	-22.8935	-42.4683	Saquarema	Área de Proteção Ambiental da Massambaba	4.1883
-22.923	-42.374	-22.8935	-42.4683		Área de Proteção Ambiental da Massambaba	10.2111
-22.9223	-42.4453	-22.8935	-42.4683		Reserva Ecológica Estadual de Jacarepiá	3.9673
-22.9202	-42.5102	-22.8935	-42.4683		Área de Proteção Ambiental da Massambaba	5.2174
-22.5055	-42.275	-22.6508	-42.3905	Silva Jardim		19.9995
-22.4903	-43.0657	-22.4161	-42.972	Teresópolis	Parque Nacional da Serra dos Órgãos	12.6701
-22.465	-43.032222	-22.4161	-42.972		Parque Nacional da Serra dos Órgãos	8.2312
-22.4627	-43.0244	-22.4161	-42.972		Parque Nacional da Serra dos Órgãos	7.4649
-22.4616	-43.0283	-22.4161	-42.972		Parque Nacional da Serra dos Órgãos	7.6795
-22.4601	-43.028	-22.4161	-42.972		Parque Nacional da Serra dos Órgãos	7.5480
-22.46	-43.0281	-22.4161	-42.972		Parque Nacional da Serra dos Órgãos	7.5487
-22.46	-43.0286	-22.4161	-42.972		Parque Nacional da Serra dos Órgãos	7.5882
-22.455	-43.025556	-22.4161	-42.972			6.9965
-22.45	-43.166667	-22.4161	-42.972		Parque Nacional da Serra dos Órgãos	20.3887
-22.4483	-42.9833	-22.4161	-42.972		Parque Nacional da Serra dos Órgãos	3.7506
-22.4166	-42.9833	-22.4161	-42.972		Parque Nacional da Serra dos Órgãos	1.1647
-22.4161	-42.972	-22.4161	-42.972			12.0948
-22.3786	-42.8055	-22.4161	-42.972			17.6406
-22.31889	-42.5345	-22.4161	-42.972		Parque Nacional da Serra dos Órgãos	46.3279
-22.4076	-43.661	-22.4076	-43.661	Vassouras		11.9868
					Mean deviation	13.8087

**Table 2.** Information for Gnaphalieae genera and species. Distribution, habitat and phenology, based on herbaria data. New or not found occurrences, based on comparison to herbarium data and Flora e Funga do Brasil (Deble et al. 2023).

Species		Distribution (Municipality)	Habitat	New Occurrence	Occurrence not Found	Phenology
***Achyrocline***	***alata*** (Kunth) DC.	Cachoeiras de Macacu, Itatiaia, Magé, Nova Friburgo, Paraty, Petrópolis, Rio Claro, Rio de Janeiro, Sapucaia and Teresópolis	Grasslands and Restinga	-	-	Jan/Feb/Mar/Apr/May/ Jun/Jul/Aug/Sept/Nov
	***arrajodoana*** Mattf.	Itatiaia, Nova Friburgo, Rio de Janeiro and Teresópolis	Grasslands	-	-	Mar/May/Jun/Jul
	***chionaea*** (DC.) Deble & Marchiori	Itatiaia, Nova Friburgo and Seropédica	Grasslands and Restinga	-	-	Jun/Dec
	***citrina*** Griseb.	-	-	-	Yes	-
	***flaccida*** (Weinm.) DC.	Angra dos Reis, Itatiaia, Petrópolis, Rio de Janeiro, Santa Maria Madalena and Teresópolis	Grasslands and Restinga	Yes	-	Jan/Feb/Mar/Apr/ Jul/Aug/Oct/Nov
	***gardneri*** (Baker) Deble & Marchiori	Itatiaia	Grasslands	Yes	-	August
	***lanosa*** (Wawra) Deble	Itatiaia	Grasslands	-	-	Jun/Jul
	***satureioides*** (Lam.) DC.	44 municipalities	Grasslands and Restinga	-	-	Jan to Dec
	***vargasiana*** DC.	Itatiaia, Nova Friburgo, Passa Três, Petrópolis and Rio de Janeiro	Grasslands and Restinga	-	-	Apr/May/Jun/Jul/Oct
	***vauthieriana*** DC.	Rio de Janeiro and Teresópolis	Grasslands	Yes	-	Jan/Feb/Mar
***Chevreulia***	***acuminata*** Less.	Campos dos Goytacazes, Itatiaia, Nova Friburgo, Petrópolis, Rio de Janeiro, Santa Maria Madalena, Seropédica, Teresópolis and Vassouras	Grasslands and Restinga	-	-	Jan to Dec
***Chionolaena***	***arbuscula*** DC.	Arraial do Cabo, Cachoeiras de Macacu, Itatiaia, Rio de Janeiro and Teresópolis	Grasslands and Restinga	Yes	-	Jan to Dec
	***capitata*** (Baker) Freire	Itatiaia, Rio Bonito, Rio de Janeiro and Teresópolis	Grasslands	-	-	Jan to Dec
	***isabellae*** Baker	Itatiaia, Rio de Janeiro and Teresópolis	Grasslands	-	-	Jan to Dec
	***latifolia*** (Benth) Baker	Itatiaia and Teresópolis	Grasslands	-	-	Jan to Dec
	***lychnophorioides*** Sch.Bip	Itatiaia	Grasslands	Yes	-	May/Jun
	***phylicoides*** (Gardner) Baker	Cachoeiras de Macacu, Nova Friburgo, Petrópolis, Rio de Janeiro and Teresópolis	Grasslands and Restinga	-	-	Jan/Feb/Mar/Apr/May/ Jun/Jul/Aug/Sep/Nov
	***wittigiana*** Baker	Itatiaia	Grasslands	Yes	-	Jan/May/Jul/Oct/Nov
***Facelis***	***retusa*** (Lam.) Sch.Bip	Duas Barras, Itatiaia, Nova Friburgo, Petrópolis and Rio de Janeiro	Grasslands	-	-	Sep/Oct/Dec
***Gamochaeta***	***americana*** (Mill.) Wedd.	Itatiaia, Macaé, Nova Friburgo, Paraty, Rio de Janeiro and Teresópolis	Grasslands and Restinga	-	-	Jan/Apr/May/Jun/Jul/ Aug/Sep/Oct/Nov/Dec
	***calviceps*** (Fernald) Cabrera	-	-	-	Yes	-
	***grazielae*** (Rizzini) Deble	Itatiaia and Rio de Janeiro	Grasslands	-	-	Jan/Nov
	***hiemalis*** Cabrera	-	-	-	Yes	-
	***nigrevestis*** Deble & Marchiori	-	-	-	Yes	-
	***pensylvanica*** (Willd.) Cabrera	Itatiaia, Rio das Ostras, Rio de Janeiro and Seropédica	Grasslands and Restinga	-	-	Apr/May/Jul/ Aug/Sep/Dec
	***purpurea*** (L.) Cabrera	Itatiaia, Mangaratiba, Petrópolis, Rio de Janeiro, Seropédica, Teresópolis and Vassouras	Grasslands and Restinga	-		Jan/Feb/Mar/May/Jul/ Aug/Sep/Oct/Nov/Dec
	***rizzinii*** Cabrera	-	-	-	Yes	-
	***simplicicaulis*** (Willd. ex Spreng.) Cabrera	Barra do Piraí, Cabo Frio, Nova Friburgo, Petrópolis, Rio de Janeiro, Sapucaia and Teresópolis	Grasslands and Restinga	-	-	Mar/Aug/Oct/Nov
	***stachydifolia*** (Lam.) Cabrera	Itatiaia and Rio de Janeiro	Grasslands and Restinga	Yes	-	Jan/Mar/Nov/Dec
***Gnaphalium***	***polycaulon*** Pers.	Nova Friburgo and Rio de Janeiro	Grasslands and Restinga	-	-	Apr/Jul/Aug/Dec
***Lucilia***	***ferruginea*** Baker	Unknown	Unknown	-	-	Oct
	***linearifolia*** Baker	Itatiaia	Grasslands	-	-	Apr/Sep/Oct
	***lycopodioides*** (Less.) S.E.Freire	Itatiaia, Rio de Janeiro and Teresópolis	Grasslands	-	-	Feb/Apr/May/ Jun/Jul/Sep/Nov
	***tomentosa*** Wedd.	Unknown	Unknown	Yes	-	Sep
***Pseudognaphalium***	***cheiranthifolium*** (Lam.) Hilliard & Burtt	Campos dos Goytacazes, Itatiaia, Macaé, Nova Friburgo, Paraty, Rio de Janeiro, Santa Maria Madalena and Teresópolis	Grasslands and Restinga	-	-	Jan to Dec
	***gaudichaudianum*** (DC.) Anderb.	Barra do Piraí, Itatiaia and Rio de Janeiro	Grasslands and Restinga	Yes	-	Jan/Jun/Aug/Oct
