# Supplementary figures and images for: Gnaphalieae (Asteraceae): diversity and distribution in Rio de Janeiro – Brazil
Source: Biodivers Data J. 2025 May 21;13:e142891. doi: 10.3897/BDJ.13.e142891 (PMC12120492; doi:10.3897/BDJ.13.e142891)

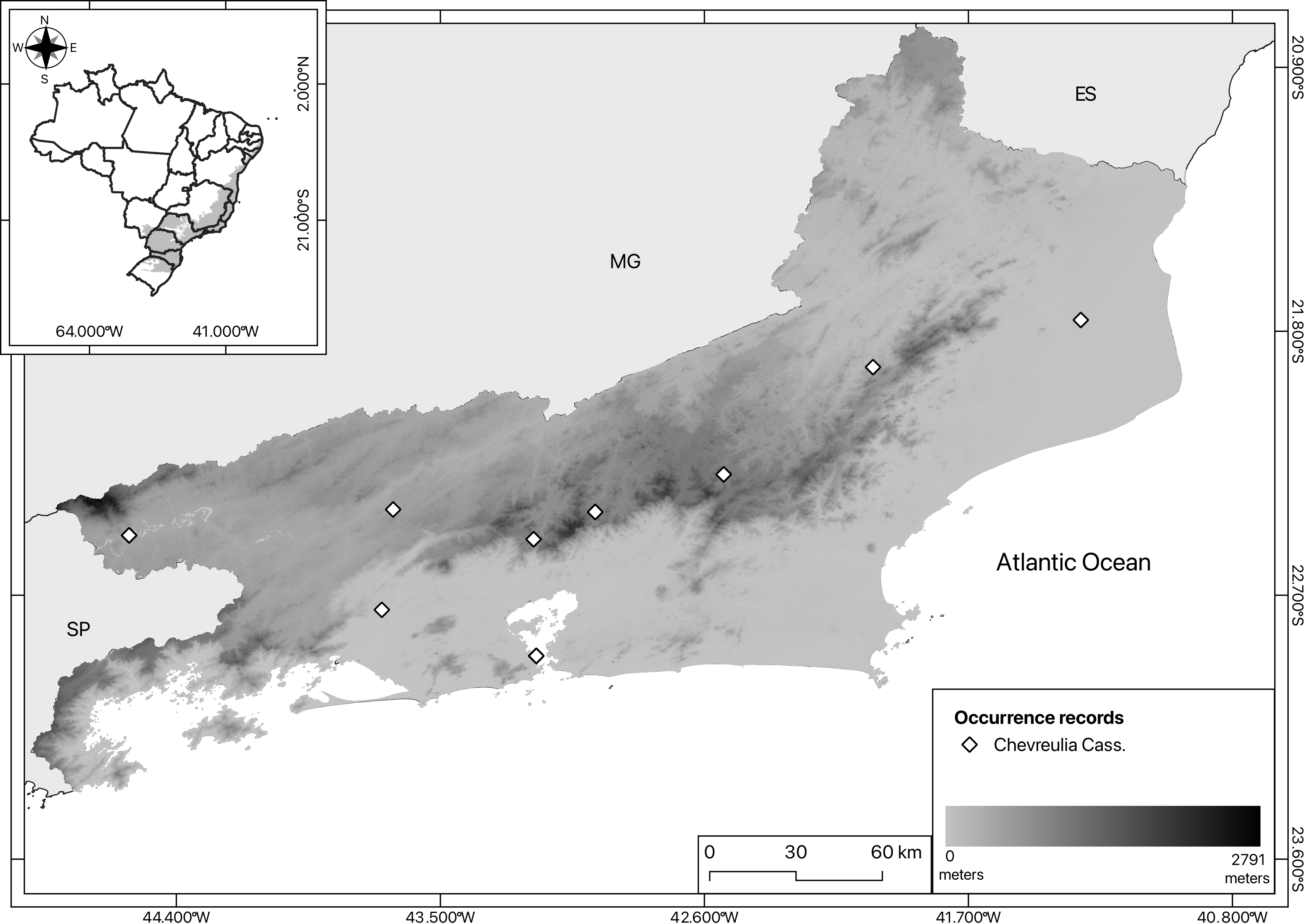

Supplement: Supplementary material 1 — Gnaphalieae distribution maps for each of the eight genera [file bdj-13-e142891-s001.zip › Supplementar_material2/Figure2_distribution_chevreulia.png]

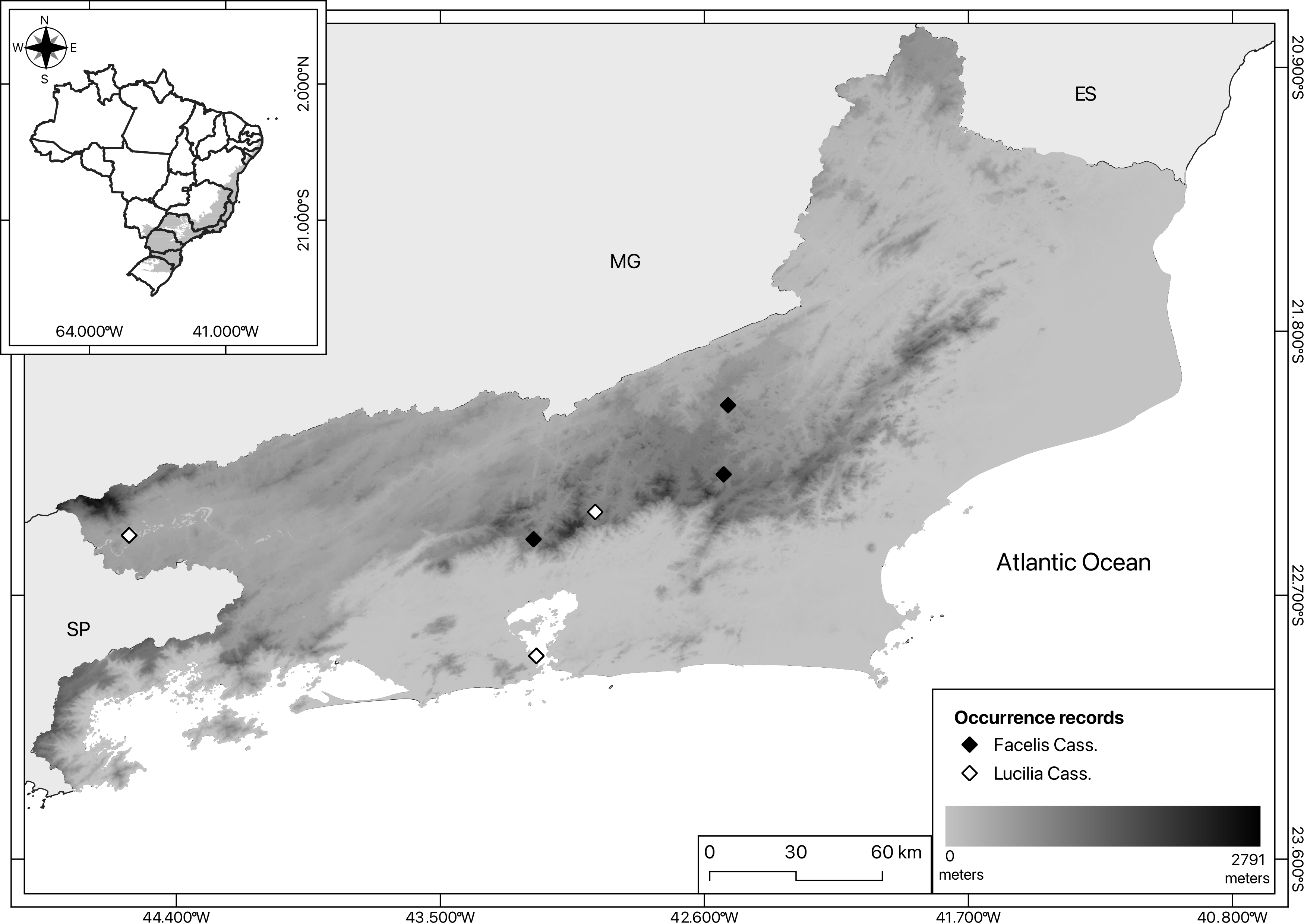

Supplement: Supplementary material 1 — Gnaphalieae distribution maps for each of the eight genera [file bdj-13-e142891-s001.zip › Supplementar_material2/Figure4_distribution_facelis_lucilia.png]

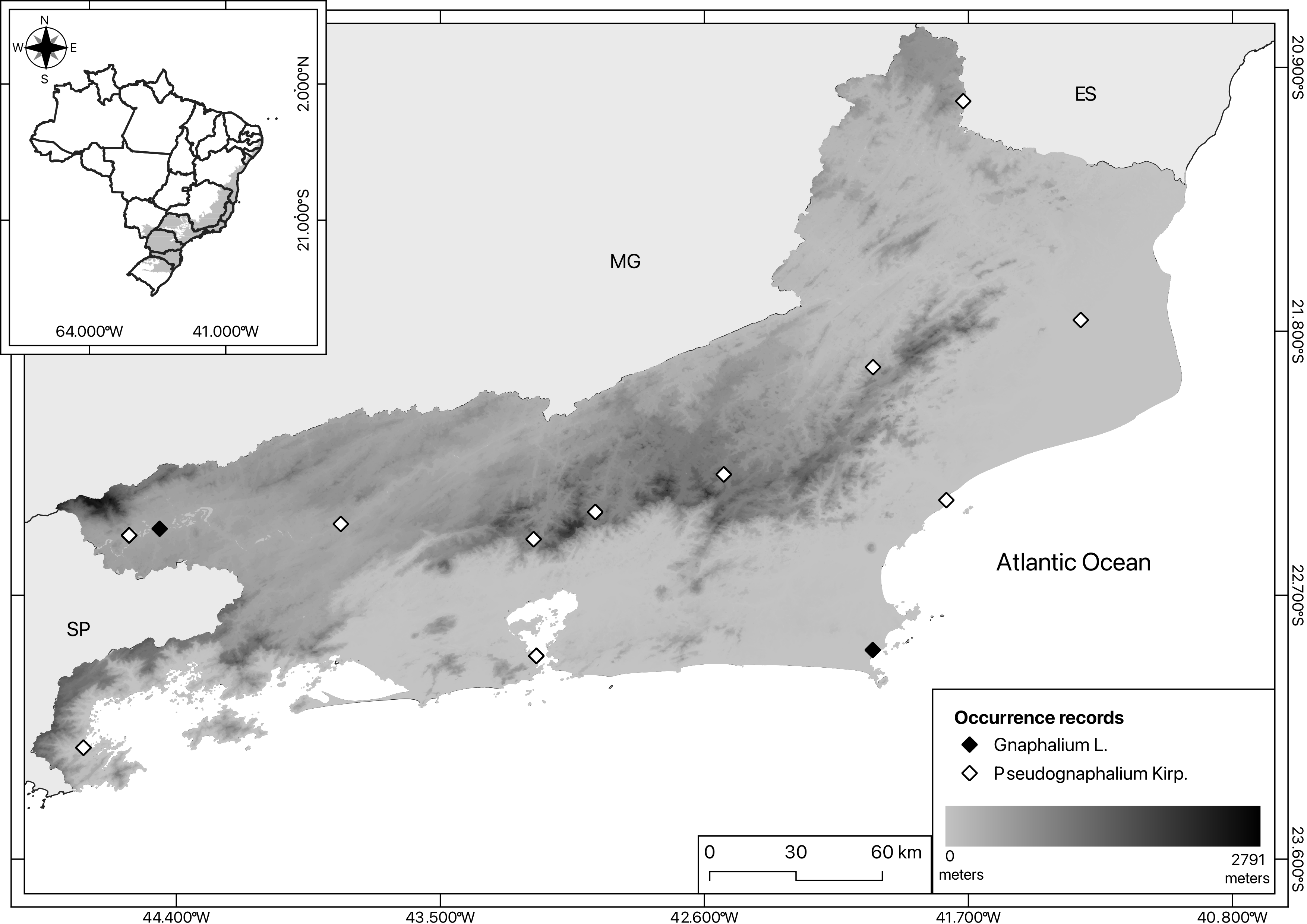

Supplement: Supplementary material 1 — Gnaphalieae distribution maps for each of the eight genera [file bdj-13-e142891-s001.zip › Supplementar_material2/Figure5_distribution_gnaphalium_pseudognaphalium.png]

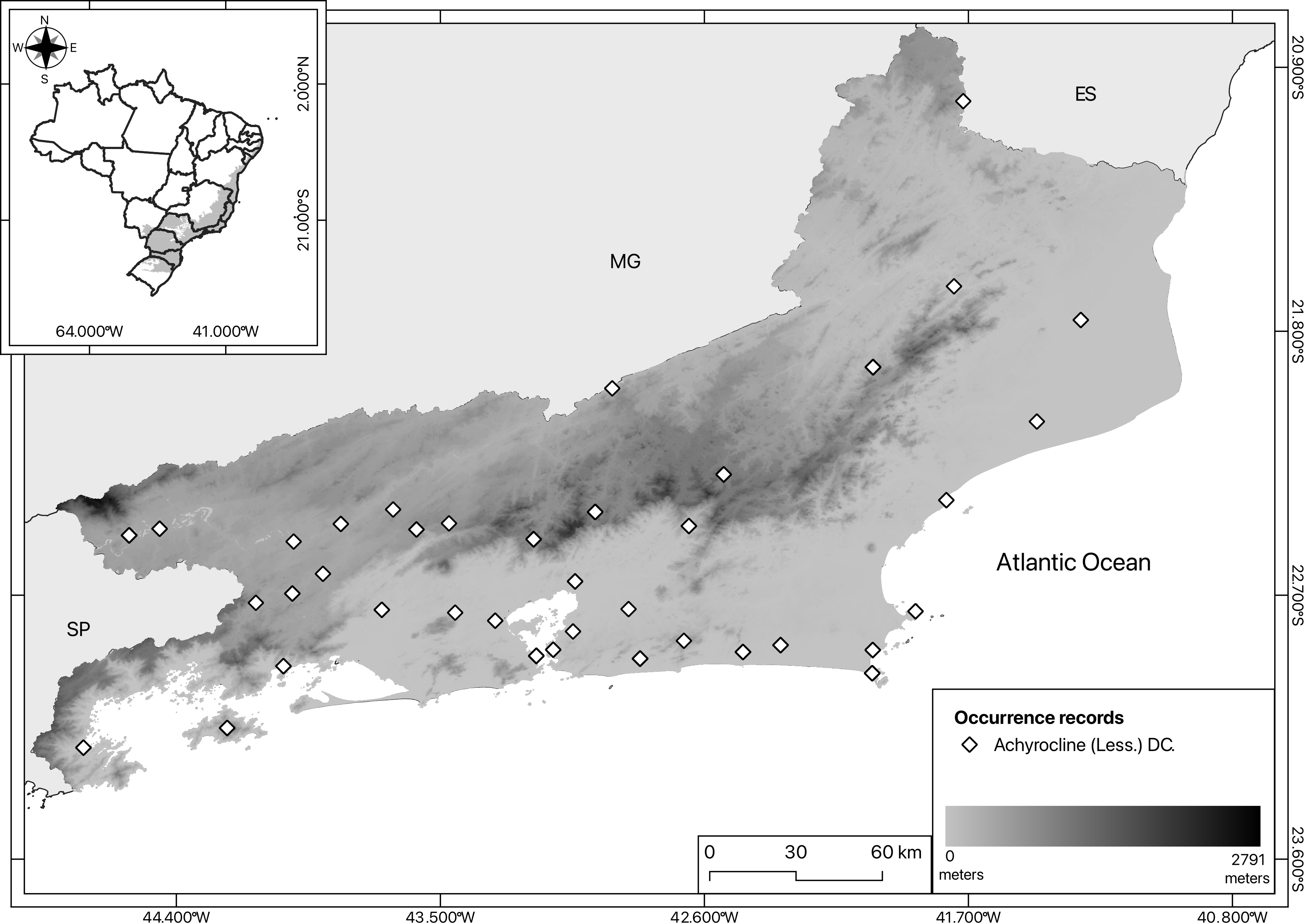

Supplement: Supplementary material 1 — Gnaphalieae distribution maps for each of the eight genera [file bdj-13-e142891-s001.zip › Supplementar_material2/Figure1_distribution_achyrocline.png]

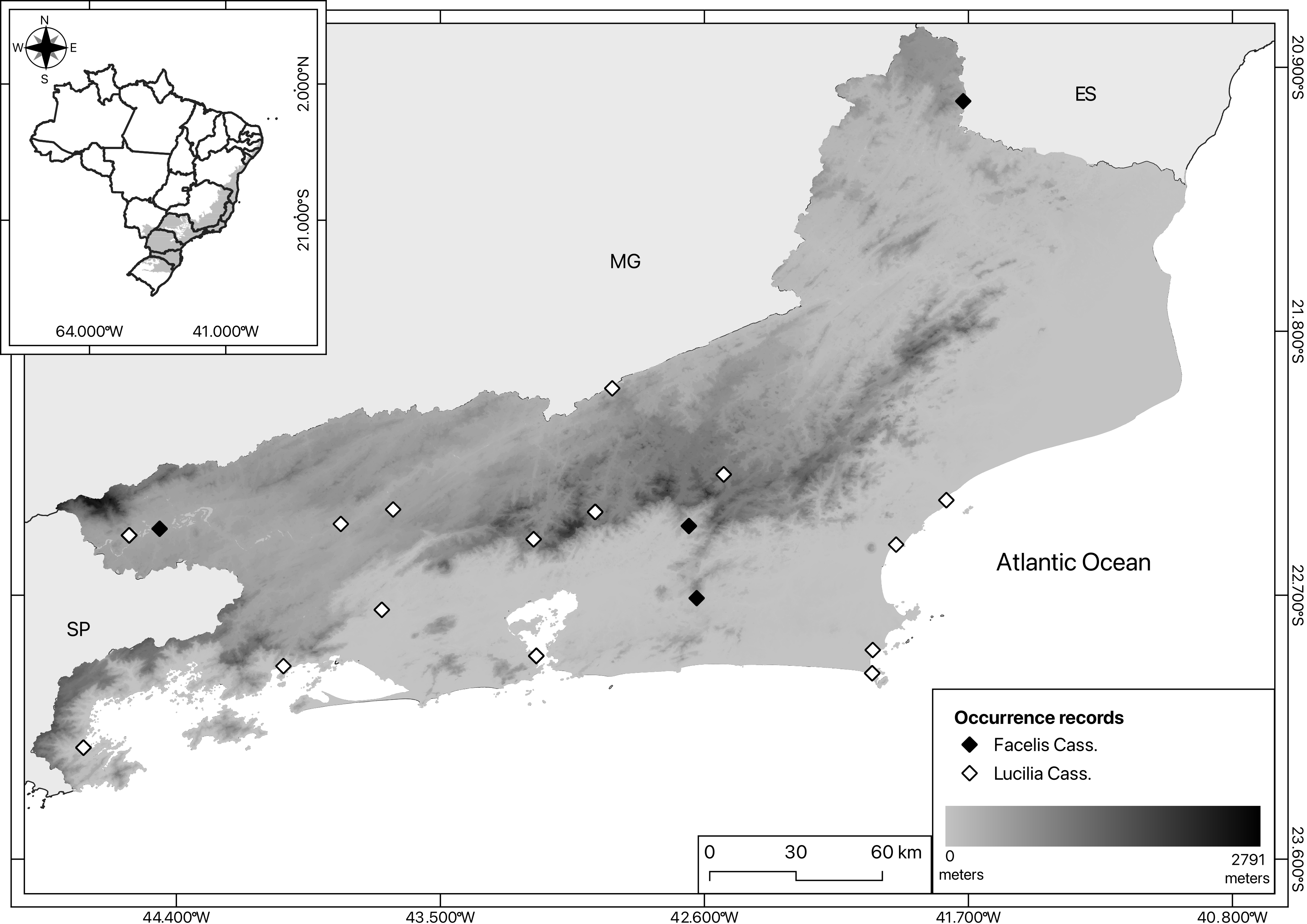

Supplement: Supplementary material 1 — Gnaphalieae distribution maps for each of the eight genera [file bdj-13-e142891-s001.zip › Supplementar_material2/Figure3_distribution_chionolaena_gamochaeta.png]
